# Prevalence of Obesity and Its Associated Comorbidities in Adults with Asthma: A Single-Center Study in Saudi Arabia

**DOI:** 10.3390/medicina60111785

**Published:** 2024-10-31

**Authors:** Abdulrhman S. Alghamdi, Khalid S. Alwadeai, Mohammed A. Almeshari, Saad A. Alhammad, Sulaiman S. Alsaif, Wael A. Alshehri, Mushabbab A. Alahmari, Turki M. Alanazi, Rayan A. Siraj, Fahad Abuguyan, Tareq F. Alotaibi, Saleh S. Algarni

**Affiliations:** 1 Department of Health Rehabilitation Sciences, College of Applied Medical Sciences, King Saud University, Riyadh 11411, Saudi Arabiashammad@ksu.edu.sa (S.A.A.); salsaif3@ksu.edu.sa (S.S.A.); 2 Department of Respiratory Therapy, King Saud University Medical City Hospital, Riyadh 12372, Saudi Arabia; walshehri3@ksu.edu.sa; 3 Department of Respiratory Therapy, College of Applied Medical Sciences, University of Bisha, Bisha 67714, Saudi Arabia; 4 Health and Humanities Research Center, University of Bisha, Bisha 67714, Saudi Arabia; 5 Department of Respiratory Therapy, King Saud bin Abdelaziz University for Health Sciences, Al Ahsa 31982, Saudi Arabia; 6 King Abdullah International Medical Research Center, Al Ahsa 31982, Saudi Arabia; 7 Department of Respiratory Care, College of Applied Medical Sciences, King Faisal University, Al-Ahsa 31982, Saudi Arabia; 8 Department of Emergency Medicine, College of Medicine, King Saud University, Riyadh 11411, Saudi Arabia; 9 Department of Respiratory Therapy, College of Applied Medical Sciences, King Saud bin Abdulaziz University for Health Sciences, Riyadh 14611, Saudi Arabia; 10 King Abdullah International Medical Research Center, Riyadh 11481, Saudi Arabia; 11 Respiratory Services, King Abdulaziz Medical City, Riyadh 11426, Saudi Arabia

**Keywords:** body mass index, diabetes mellitus, cardiovascular disease, allergic rhinitis, sinusitis, gastroesophageal reflux disease, obstructive sleep apnea

## Abstract

*Background and Objectives*: Asthma is associated with several comorbidities, one of which is obesity. The worldwide increase in obesity has been accompanied by a parallel rise in asthma prevalence, with obesity recognized as a significant risk factor for both the development and severity of asthma. Obesity is often linked to various comorbidities, which can complicate asthma management and lead to poorer clinical outcomes. This study aims to investigate the prevalence of obesity and its comorbidities in adults with asthma in a single center in Saudi Arabia, providing an overview of the associated health implications. *Materials and Methods*: This single-center, retrospective study aimed to assess the prevalence of obesity and other comorbidities in asthma patients. Data were collected from King Khalid University Hospital in Saudi Arabia between July 2023 and December 2023. *Results*: This study revealed that 72.1% of asthma patients were either obese or overweight. Female patients had significantly higher BMI values compared to males. Our study revealed that 38.21% of female asthma patients (mean age = 57 ± 13.85 years) had comorbidities compared to 24.14% of male asthma patients (mean age = 59 ± 14.02 years). Furthermore, the proportion of obese asthmatic patients with comorbidities was significantly greater than those without comorbidities. *Conclusions*: This study investigates obesity prevalence and associated comorbidities in adult asthmatics in a single center in Saudi Arabia. The findings reveal a 72.1% rate of obesity and overweight among asthmatic patients, with higher BMI and comorbidity prevalence in females. These results underscore the need for targeted interventions addressing obesity and comorbidities, especially in female asthmatics.

## 1. Introduction

Asthma is a chronic inflammatory disease of the airways characterized by recurrent episodes of wheezing, chest tightness, shortness of breath, and coughing [1,2]. The disease burden of asthmatic patients with obesity is significantly exacerbated, intensifying symptoms and contributing to a complex interplay of comorbidities that complicate management and quality of life. In Saudi Arabia, the nationally weighted prevalence of obesity, defined at a BMI of ≥30, is 24.7%, with studies showing a significantly higher prevalence of 46% among asthmatic patients [3,4]. Several comorbid conditions are associated with both asthma and obesity, including allergic rhinitis, sinusitis, gastroesophageal reflux disease, diabetes mellitus, cardiovascular diseases, and obstructive sleep apnea (OSA) [5]. In addition, asthma itself may contribute to the development or worsening of these comorbidities [6].

Research has established a strong link between obesity and asthma [7,8], showing that obesity worsens asthma symptoms, reduces lung function, and increases the risk of hospitalization [9]. Several mechanisms, such as restricted airflow due to excess weight and heightened systemic inflammation, could explain this relationship [10,11,12]. Despite this, significant gaps remain in our understanding of obesity and its comorbidities among adult asthmatics in Saudi Arabia, especially since many studies rely on self-reported data.

Obesity is a key risk factor for asthma outcomes, contributing to poor asthma control, increased hospitalization rates, and a higher risk of exacerbations [13,14]. Additionally, obesity is frequently associated with comorbidities such as diabetes and cardiovascular diseases, further complicating asthma management and worsening clinical outcomes [15,16,17]. Psychological conditions, including anxiety and depression, are also more common in obese asthma patients, compounding the severity of their condition and reducing adherence to treatment [18,19]. Evidence suggests that asthma, its comorbidities, and obesity are more prevalent and severe in women than in men [20]. Addressing obesity in asthma patients is crucial not only for improving asthma control but also for reducing the burden of associated comorbidities, thereby enhancing overall quality of life.

This study aims to fill knowledge gaps by investigating the prevalence of obesity and its associated comorbidities in adult asthmatics at a single center in Saudi Arabia, with an emphasis on gender differences. By employing objective measures of body mass index (BMI) and evaluating comorbidities, this study can provide valuable insights into the disease burden faced by this population. These findings will inform the development of targeted interventions and improve overall asthma management strategies.

## 2. Materials and Methods

### 2.1. Study Design and Settings

This single-center, retrospective study was conducted to investigate the prevalence of obesity and comorbidities among patients with asthma. The data collection process was carried out between 23 July 2023 and 23 December 2023 at King Khalid University Hospital in Saudi Arabia. This study took place at King Khalid University Hospital, which is a tertiary academic center located in Riyadh, Saudi Arabia. It primarily serves the local population in Riyadh and its surrounding regions. The hospital serves a diverse population, including Saudi citizens and residents. The data collection process was carried out between 23 July 2023 and 23 December 2023. The database comprises clinical and demographic data. Clinical data include diagnoses and other comorbidities such as cardiovascular diseases, diabetes, respiratory illnesses, and psychological disorders. The data used in this study are derived from a local electronic database with its own specific characteristics. Data validation was performed through automated and manual checks to ensure accuracy and completeness. The data are stored on secure servers, with access restricted to authorized personnel, in compliance with data protection regulations.

### 2.2. Study Population

**Figure 1:**
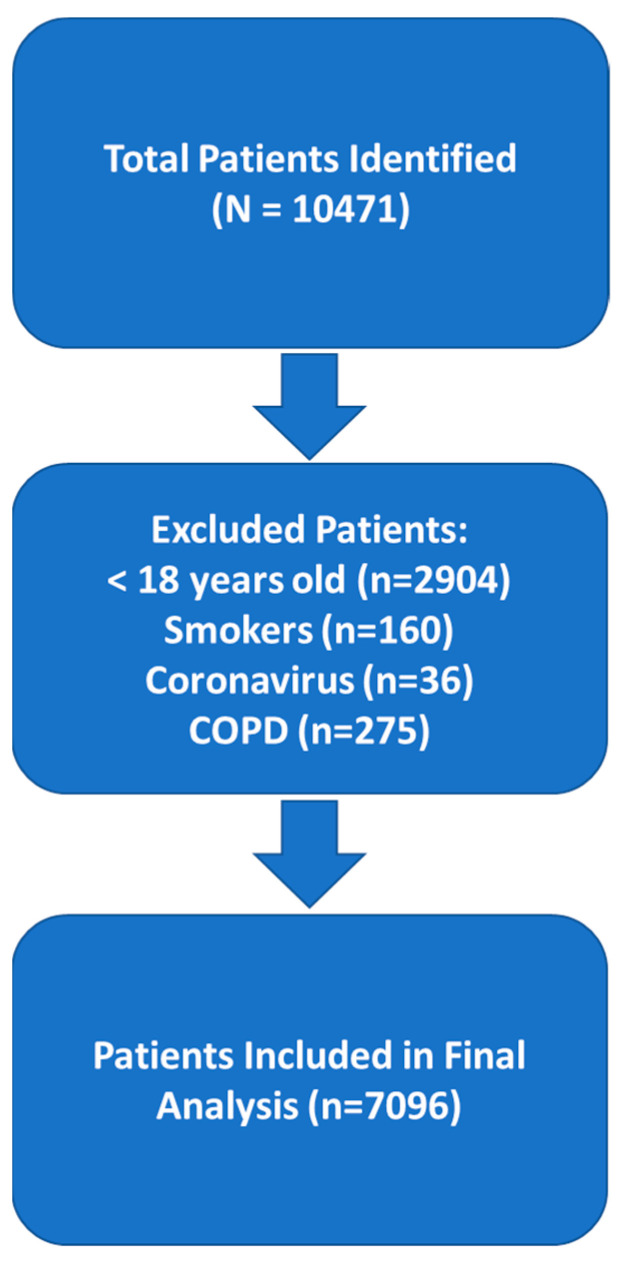
Flowchart of patient selection and exclusion criteria.

We conducted a retrospective review of electronic medical records for 10,471 patients with confirmed asthma diagnoses who had scheduled visits and consultations with specialists. The data collection spanned from January 2022 to October 2023. We collected demographic data (e.g., height, weight, BMI, and age and gender). In this study, we included only patients who had been diagnosed with asthma by a multidisciplinary team and whose diagnosis was based on current national (The Saudi Initiative for Asthma) and international (The Global Initiative for Asthma) accepted criteria, which are based on a clinical assessment by a detailed history and physical examination supported by spirometry with reversibility testing [1]. In this center, patients with comorbidities are diagnosed in accordance with the Evidence-Based Clinical Practice Guidelines. Patients with asthma who had only one comorbid condition were included in this study. It is noteworthy that the starting point for collecting data from the electronic chart was established to be the first 24 h of admission. In the final analysis, we only included asthmatic patients aged 18 and above, as well as patients without smoking history, due to the difficulty of separating asthma from chronic obstructive pulmonary disease in smokers (Figure 1). Smoking can exacerbate asthma symptoms and complicate the interpretation of asthma-related outcomes. Excluding smokers helps to minimize these confounding variables, thereby providing clearer insights into asthma management in non-smoking individuals. COVID-19 can cause respiratory symptoms and complications that overlap with or exacerbate asthma, potentially confounding the study results. The inclusion of patients with COVID-19 could skew the data and make it difficult to attribute observed effects solely to asthma. Chronic obstructive pulmonary disease (COPD) has distinct pathophysiological features and management strategies compared to asthma. Including patients with COPD would introduce significant heterogeneity, as these patients may have different responses to treatment and disease progression.

### 2.3. Body Mass Index

Height and weight were routinely measured in the clinics, with patients barefoot and wearing light clothing, using a medical scale supervised by a trained nurse. We only collected BMI values based on the heights and weights measured. All asthmatic patients included in the current study were divided into five groups in accordance with the Centers for Disease Control and Prevention (CDC) classifications: (1) patients with BMI values of 18.5 to 24.9 kg/m^2^ (lean or normal weight); (2) patients with BMI values of 25 to 29.9 kg/m^2^ (overweight); (3) patients with BMI values of 30 to 34.9 kg/m^2^ (mild or class I obesity); (4) patients with BMI values of 35 to 39.9 kg/m^2^ (moderate or class II obesity); and (5) patients with BMI values of 40 kg/m^2^ or above (morbid or class III obesity) [5,6].

### 2.4. Ethical Considerations

Prior to the start of this study, ethical approval (Project No. E-23-7978) was obtained from the Institutional Review Board at the Faculty of Medicine at King Saud University, Saudi Arabia.

### 2.5. Statistical Analysis

Descriptive statistics were utilized to summarize the study data. For categorical variables, frequencies and percentages were calculated. Continuous variables were summarized using mean and standard deviation (SD). For inferential statistics, comparisons between groups were conducted using appropriate tests based on the type of variable. The chi-square test was employed for the comparison of categorical variables, while the independent-sample *t*-test was used for continuous variables. A *p*-value of less than 0.05 was considered statistically significant. All analyses were performed using STATA v.18, and the graph was prepared using GraphPad Prism v.10.

## 3. Results

### 3.1. Patient Characteristics

In total, 10,471 subjects with confirmed asthma diagnoses were identified from the databases from January 2022 to October 2023. After excluding patients < 18 years old (*n* = 2904), smokers (*n* = 160), those with COVID-19 (*n* = 36), and those with COPD (*n* = 275), a total of 7096 asthma patients met our inclusion criteria and were included in the final analysis.

The mean ± SD age of the study population was 56 ± 11 years, and there were more females (58.85%) than males. Of the 7096 included patients, 11.8% had a BMI of less than 18.5 kg/m^2^ (underweight), 16.1% had BMIs between ≥18.5 and <25 kg/m^2^ (normal weight), 24.0% had BMIs between ≥25 and <30 kg/m^2^ (overweight), and 48.1% had BMIs of ≥30 kg/m^2^ (obese I = 22.6%, obese II = 14.1%, and obese III = 11.4%). The prevalence of obesity and overweight in patients with asthma was 72.1%. The BMI was statistically significantly greater in females than in males (mean = 32.34 ± 8.17 vs. 29.07 ± 7.61; *p* < 0.001) (Table 1).

### 3.2. Asthmatic Patients with Comorbidities

This study has found that 2301 asthmatic patients have another comorbidity, representing 32.42% of the total patients included in this study. The comorbidities that we found are as follows: gastroesophageal reflux disease, respiratory illness (allergic rhinitis, bronchiectasis, and sinusitis), cardiovascular diseases (atrial fibrillation, congestive heart failure, ischemic heart disease, and hypertension), diabetes mellitus, and sleep apnea, which are listed in Table 2. Approximately 25.77% of total comorbidities are caused by cardiovascular diseases, 23.78% are caused by respiratory illness, 17.10% are caused by psychological disorders, and 16.86% are caused by diabetes mellitus.

### 3.3. Body Mass Index Comparison: Pure Asthma Versus Asthma with Comorbidities

We have shown no statistically significant difference between asthmatic patients without (mean = 32.83 ± 8.53) and asthmatic patients with comorbidities (mean = 33.01 ± 7.53) in terms of BMI. However, our results revealed a statistically significant higher BMI in obese males and obese females with comorbidities compared to those without comorbidities (*p* = 0.0103 and *p* = 0.00046, respectively) (Table 3).

### 3.4. Gender and Age Differences in Asthma Patients with Comorbidities

Our study showed that females with asthma with comorbidities represent 36.46% (age mean = 57 ± 13.85) of the total female patients with asthma, while males represent 24.33% (age mean = 59 ± 14.02) of the total males with asthma. Asthmatic females with comorbidities are significantly higher than males (*p* = 0.002) (Table 4).

### 3.5. Body Mass Index Variations Among Asthmatic Patients Admitted to the Emergency Room with or Without Comorbidities

**Figure 2:**
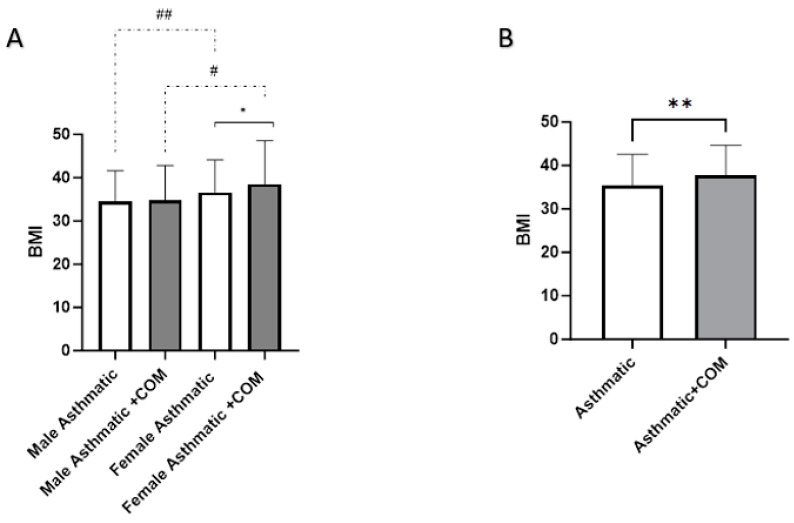
BMI variations in asthmatic patients with and without comorbidities. Patients were divided into four groups (**A**) based on body mass index (BMI) and presence of comorbidities: obese male asthmatics, obese male asthmatics with comorbidities, obese female asthmatics, and obese female asthmatics with comorbidities. (**B**) illustrates another comparison of obese asthmatics with comorbidities with obese asthmatics without comorbidities. Each data point represents mean ± SD. * *p* < 0.05 and ** *p* < 0.01, # *p* < 0.05, and ## *p* < 0.01. +COM = with comorbidities.

In our study, we investigated the relationship between BMI variations and asthma exacerbations among patients admitted to the ER, considering the presence or absence of comorbidities. The study population included asthmatic patients admitted to the ER with documented exacerbations, with data collected on BMI, gender, and comorbidities. Emergency visits were recorded using a standardized patient database, and comorbidities were identified through medical records and categorized accordingly. The analysis revealed that female asthmatics without comorbidities had a significantly higher BMI compared to males (mean = 36.62 ± 7.46 vs. 34.54 ± 7.03, *p* < 0.01). Similarly, female asthmatics with comorbidities also exhibited a significantly higher BMI than their male counterparts (mean = 38.56 ± 9.94 vs. 34.83 ± 8.00, *p* < 0.01). Furthermore, the proportion of obese (BMI > 30) asthmatic patients with comorbidities was significantly greater than that of obese asthmatic patients without comorbidities (*p* < 0.001) (Figure 2). These findings suggest that female asthmatics, irrespective of comorbidities, who are admitted to the ER due to asthma exacerbations tend to have a higher BMI. The increased prevalence of obesity among asthmatics with comorbidities indicates a possible interaction between obesity and additional health conditions, necessitating further investigation.

## 4. Discussion

In our study, 72.1% of patients with asthma were obese or overweight. Females had significantly higher BMIs than males. Furthermore, our findings revealed that obese asthmatic patients with comorbidities were considerably more prevalent than those without. Obese males and obese females with comorbidities had significantly higher BMIs than those without. In addition, asthmatic females showed a notably higher rate of comorbidities than males. These findings suggest that female asthmatic patients are at higher risk of obesity and other comorbidities. To our knowledge, this is the first study investigating the prevalence of obesity and its associated comorbidities in adult asthmatics from a single center in Saudi Arabia.

Obesity is a well-established risk factor for asthma and other diseases such as hypertension, diabetes mellitus, and ischemic heart disease [21]. Obesity can lead to asthma exacerbations and hospitalization. Our results showed that asthmatic patients who were overweight (25%) or obese (48.1%) were prevalent, which is consistent with previous research. It has been reported that obesity prevalence in the Riyadh region was 26.9%, making it the second-highest region in terms of obesity prevalence, following the Eastern Region, which had a rate of 29.4%. A study conducted in Saudi Arabia reported an obesity prevalence of 46% among asthmatic patients [4], while a study in the Netherlands found a prevalence of 52% [22]. It is not surprising that individuals with chronic diseases and certain medical conditions experience a higher prevalence of obesity than the general population due to, for instance, chronic inflammation, which can disrupt normal metabolic processes and lead to weight gain and obesity [23] or medication use [24]. In contrast, other studies show a lower rate of obesity among asthmatic patients [25,26]. This discrepancy may be attributed to the fact that asthmatic patients in the aforementioned studies were based on self-reported asthma. In our study, we included only patients with a confirmed asthma diagnosis based on national and international criteria. BMI was calculated using clinic-measured height and weight, with patients barefoot and in light clothing, under the supervision of a trained nurse, enhancing our study’s reliability. Moreover, the percentage of obesity has increased among the general population in Saudi Arabia [3], which may justify the high prevalence of obesity in our findings. Nevertheless, the exact relationship between asthma and obesity remains unclear. Previous research suggested that asthma control therapies, such as corticosteroids, may contribute to obesity [27,28]. On the other hand, numerous studies have considered obesity to be a risk factor for asthma [29], showing that patients with obesity are more likely to develop this condition. A novel disease phenotype, obesity-associated asthma, may develop as a result, requiring careful evaluation and management. Further studies are needed to better understand the characteristics and underlying mechanisms of this phenotype.

We found that approximately 32% of patients with asthma have another comorbidity, which is consistent with previous evidence demonstrating higher rates of comorbidities associated with asthma [30,31,32,33]. For instance, our study showed that 25.77% of total comorbidities were caused by cardiovascular diseases, which is in line with a previous study that found that patients with asthma had a 42% higher risk of CVD than patients without asthma [34]. However, this study indicated that CVD risk is more pronounced in uncontrolled asthma, which may explain the variation in the findings. In our study, diabetes mellitus represented around 16.86% of the comorbidities, which is supported by Brumpton et al., who found a 43% increase in the risk of incident asthma in people with type 2 diabetes after monitoring them for 11 years [35]. The link between asthma and obesity and its associated comorbidities may be due to systemic inflammation or, at least, to specific asthma endophenotypes [36]. Systemic chronic inflammation contributes to endothelial dysfunction, atherosclerosis, and cardiovascular disease [37]. Asthma, as an inflammatory condition, is similarly associated with elevated systemic inflammatory markers [38]. A 2022 meta-analysis linked asthma to increased all-cause and cardiovascular mortality [15]. In individuals with asthma, a higher BMI is closely linked to increased oxidative stress and elevated levels of interleukin-6, along with other proinflammatory markers [39,40]. The coexistence of asthma and obesity may synergistically increase levels of circulating proinflammatory cytokines, further increasing the likelihood of insulin resistance and type 2 diabetes mellitus [41]. This heightened inflammatory state may be a key factor in the pathogenesis of both conditions, with asthma and obesity potentially reinforcing each other through interconnected proinflammatory pathways. Given the observed synergy between obesity and comorbidities in asthma, it is important to implement early interventions that target obesity as a modifiable risk factor for other comorbidities. However, the lack of population-specific data on comorbidities in asthmatic patients in Saudi Arabia underscores the need for future local research, although we have compared our findings with global studies for context.

In our study, BMI was associated with asthma and found to be significantly higher in females than in males, which is consistent with previous findings [42,43,44]. For example, a longitudinal study investigating the strength of the association between metabolic syndrome and BMI with incident asthma in adults found a strong link between BMI and asthma, specifically among women [42]. In line with our findings, women with asthma tend to have higher rates of obesity, resulting in a distinct neutrophilic asthma phenotype associated with greater morbidity and reduced responses to standard treatments like inhaled corticosteroids [14,45]. Asthma symptoms are compounded by the influence of female sex hormones such as estrogen and progesterone, particularly during menstrual cycles, pregnancy, and menopause [20]. Additionally, gender-specific behaviors such as physical inactivity in women and smoking in men exacerbate asthma outcomes, highlighting the need for tailored interventions. Our study indicated that females with asthma had significantly more comorbidities than males. Our results are consistent with previous studies showing that gender differences may contribute to other comorbidities, such as CVD, depression, and respiratory illness [36,46,47]. Nevertheless, a previous study showed that males with asthma had a higher prevalence of COPD than females, which could be explained by the number of active smokers among these male patients [48]. This evidence underscores the necessity of addressing gender differences in clinical research and asthma care to optimize treatment outcomes. A holistic approach that includes behavioral, hormonal, and physiological factors is essential for reducing disparities and improving the quality of life for asthma patients across genders.

Our findings revealed that female asthmatics with or without comorbidities admitted to the ER due to asthma exacerbations have significantly higher BMIs than males. Our findings align with previous research showing significantly higher rates of emergency visits and hospitalizations among females [49], a pattern also seen in severely obese patients who experience a higher prevalence of uncontrolled asthma. On the other hand, other studies found no association between BMI and ER admission [50,51,52,53]. These inconsistent findings may be due to the methodologies used in previous studies. It has also been suggested that BMI alone is not an accurate indicator of body fat mass [54]. Moreover, there is a complex relationship between metabolic dysregulation and asthma exacerbation. An earlier meta-analysis reported no significant sex differences in BMI concerning the odds of developing incident asthma; however, there was a dose-dependent increase in risk [13]. These inconsistent results may be due to differences in sex hormones, such as leptin levels and the potential role of estrogen in asthma expression in individuals with obesity. Therefore, future studies should explore the pathways involved in this effect.

Treating and controlling asthma effectively can help reduce the risk of obesity and related comorbidities. Improved asthma control can lead to increased physical activity levels, which aid in weight management and reduce obesity. Additionally, managing asthma can lower systemic inflammation and stress in the body, which are linked to other comorbidities such as diabetes and cardiovascular disease [55]. While controlling asthma can improve overall health and potentially lower these risks, addressing obesity and related comorbidities is essential. Improving overall health requires a comprehensive approach that includes asthma management, lifestyle changes, and treatment for obesity and its comorbidities. Addressing obesity and other comorbidities can also improve asthma symptoms. Obesity and related comorbidities can worsen asthma by increasing inflammation and negatively impacting lung functions [4]. Therefore, managing weight, improving diet, and treating other health issues can reduce inflammation and improve lung functions, leading to better asthma control. Consequently, a holistic approach that treats both asthma and associated conditions can significantly enhance asthma symptoms and overall quality of life.

### Strengths and Limitations

This study possesses several notable strengths. Firstly, unlike most studies that assess the prevalence of overweight individuals, obesity, and comorbidities in asthma patients with self-reported asthma, our research exclusively included patients diagnosed with asthma based on current national and international criteria. Secondly, while some previous studies depended on self-reported height and weight, our study calculated BMI using measurements taken with medical scales in clinics supervised by a trained nurse.

This study has several limitations. Firstly, although asthma control therapies like inhaled corticosteroids are known to improve symptoms and lung functioning, we did not account for specific doses, patient adherence, or additional therapies, which can significantly impact asthma management. Therefore, future studies using more comprehensive datasets will allow for a deeper understanding of these critical factors. Furthermore, our cross-sectional design limits our ability to establish causal relationships between asthma, obesity, and other comorbidities. Additionally, this study was conducted at a single center in Saudi Arabia, limiting the generalizability of our findings to other chronic conditions. The observational nature of this study does not establish causality between BMI and asthma exacerbations, emphasizing the need for future longitudinal studies investigating the factors influencing emergency visits and comorbidities in obese or overweight asthmatic patients. Lastly, although we used BMI as a common and accessible measure of body composition, it does not distinguish between fat and lean mass, which can have different health outcomes. Future research incorporating measures such as DXA or BIA could provide a more detailed understanding of the relationship between body composition and asthma.

## 5. Conclusions

This study found that a high proportion of asthmatic patients are obese or overweight. Furthermore, comorbid conditions were more prevalent in asthmatic females than their male counterparts. This study also demonstrated a significantly higher body mass index (BMI) among obese males and females with comorbidities compared to their counterparts without comorbidities. Notably, the BMI of female asthmatics was significantly higher than that of males, indicating a gender disparity in obesity rates. Overall, our findings underscore the need for targeted interventions to address obesity and its comorbidities, particularly among female asthmatic patients in Saudi Arabia. Comprehensive management strategies that consider both asthma control and weight management may be essential to improving health outcomes for this population.

## Figures and Tables

**Table 1:** Patient characteristics. This table summarizes the demographic and BMI characteristics of 7096 asthma patients.

Characteristic	Value
Total asthma patients included	7096
Mean age ± SD (years)	56 ± 11
Females (%)	58.85%
Males (%)	41.15%
Underweight (BMI < 18.5 kg/m^2^)	11.8%
Normal weight (BMI ≥ 18.5–<25 kg/m^2^)	16.1%
Overweight (BMI ≥ 25–<30 kg/m^2^)	24.0%
Obese I (BMI ≥ 30 kg/m^2^)	22.6%
Obese II (BMI ≥ 35–<40 kg/m^2^)	14.1%
Obese III (BMI ≥ 40 kg/m^2^)	11.4%
Total obesity prevalence (%)	72.1%
Mean BMI (females)	32.34 ± 8.17
Mean BMI (males)	29.07 ± 7.61
BMI statistical significance (*p*-value)	<0.001

**Table 2:** This table displays the number and percentage of comorbidities associated with asthma identified in our study. The comorbid conditions include gastroesophageal reflux disease (GERD), various respiratory illnesses (such as allergic rhinitis, bronchiectasis, and sinusitis), cardiovascular diseases (including atrial fibrillation, congestive heart failure, ischemic heart disease, and hypertension), diabetes mellitus, and sleep apnea.

Comorbidity	Total Count	Non-Obese Female	Non-Obese Male	% of Total Non-Obese Patients	% of Total Patient	Obese Female	Obese Male	% of Total Obese Patients	% of Total Patient
Gastroesophageal reflux	244	7	4	2.61	0.48	162	71	12.39	10.13
Allergic rhinitis	419	45	37	19.48	3.56	246	91	17.93	14.65
Sinusitis	88	10	6	3.80	0.70	51	21	3.83	3.13
Bronchiectasis	40	10	3	3.09	0.56	18	9	1.44	1.17
Hypertension	348	48	49	23.04	4.22	193	58	13.35	10.91
Congestive heart failure	121	5	8	3.09	0.56	79	29	5.74	4.69
Ischemic heart disease	71	7	2	2.14	0.39	42	20	3.30	2.69
Atrial fibrillation	53	3	2	1.19	0.22	34	14	2.55	2.09
Diabetes mellitus	388	114	33	34.92	6.39	155	86	12.82	10.47
Depression	204	2	3	1.19	0.22	139	60	10.59	8.65
Anxiety	189	3	4	1.66	0.30	128	54	9.68	7.91
Sleep apnea	136	11	5	3.80	0.70	83	37	6.38	5.22
Total	2301	265	156	100.00	18.30	1331	549	100.00	81.70

**Table 3:** This table compares the body mass index (BMI) of asthmatic patients with and without comorbidities, presenting the mean, standard error, standard deviation, and 95% confidence intervals for each group.

Group	N	Mean	Std. Err.	Std. Dev.	[95% Conf.	Interval]
Obese male asthmatics comorbidities	549	34.83 *	0.3	7.03	34.24	35.42
Non-obese male asthmatics with comorbidities	156	26.54	0.43	5.32	25.71	27.37
Obese female asthmatics with comorbidities	1331	38.56 **	0.27	9.94	38.03	39.09
Non-obese female asthmatics with comorbidities	265	24.39	0.43	6.92	23.56	25.22
Obese male asthmatics without comorbidities	660	33.74	0.3	7.67	33.15	34.33
Obese female asthmatics without comorbidities	874	36.44	0.54	8.72	35.97	36.91
Total asthmatics with comorbidities	2301	33.01	0.43	7.52	32.16	33.86
Total asthmatics without comorbidities	4795	32.81	0.14	8.52	32.53	33.1
Combined	7096	32.83	0.13	8.45	32.56	33.09
Diff		−0.2	0.45		−1.17	0.79

Note. Two-sample *t*-test with equal variances; * *p* < 0.05, ** *p* < 0.01 indicate statistical significance compared to the corresponding group without comorbidities.

**Table 4:** Morbidity is associated with females. This table illustrates the distribution of comorbidities among obese and non-obese male and female asthmatic patients, highlighting the percentage of patients with and without comorbidities within each gender.

Morbidity	Obese Females	Obese Males	Non-Obese Females	Non-Obese Males	Total
No morbidity	874	660	1706	1555	4795
%	18.23%	13.76%	35.58%	32.43%	100.00%
Have morbidity	1331	549	265	156	2301
%	57.84%	23.86%	11.52%	6.78%	100.00%
Total	2205	1209	1971	1711	7096

Note: Pearson’s chi-square test statistic = 9.9963, *p*-value = 0.002.

## Data Availability

This study’s supporting data are available from the corresponding author upon reasonable request.
